# Supplementary material for: HIVprotI: an integrated web based platform for prediction and design of HIV proteins inhibitors
Source: J Cheminform. 2018 Mar 9;10:12. doi: 10.1186/s13321-018-0266-y (PMC5845081; doi:10.1186/s13321-018-0266-y)
Supplement: Supplementary file 2 — Additional file 2. Source code of HIVProtI web server. [file 13321_2018_266_MOESM2_ESM.zip › HIVprotI_Source-code/hivprotI/weka-3-6-6/doc/weka/attributeSelection/HoldOutSubsetEvaluator.html]

HoldOutSubsetEvaluator


---


|  |  |  |
| --- | --- | --- |
| **Overview** \| **Package** \| **Class** \| **Tree** \| **Deprecated** \| **Index** \| **Help** \| **Weka's home** | |  |
| **PREV CLASS**   **NEXT CLASS** | **FRAMES**    **NO FRAMES**     **All Classes** |  |
| SUMMARY: NESTED \| FIELD \| CONSTR \| METHOD | DETAIL: FIELD \| CONSTR \| METHOD |  |


---


## weka.attributeSelection Class HoldOutSubsetEvaluator

```
java.lang.Object
  weka.attributeSelection.ASEvaluation
      weka.attributeSelection.HoldOutSubsetEvaluator
```

**All Implemented Interfaces:**: java.io.Serializable, SubsetEvaluator, CapabilitiesHandler, RevisionHandler

**Direct Known Subclasses:**: ClassifierSubsetEval

---

``` public abstract class HoldOutSubsetEvaluator extends ASEvaluation implements SubsetEvaluator ```

Abstract attribute subset evaluator capable of evaluating subsets with
respect to a data set that is distinct from that used to initialize/
train the subset evaluator.

**Version:**
:   $Revision: 1.7 $

**Author:**
:   Mark Hall (mhall@cs.waikato.ac.nz)

**See Also:**: Serialized Form

---

**Constructor Summary**

`HoldOutSubsetEvaluator()`


| **Method Summary** | |
| --- | --- |
| `abstract  double` | `evaluateSubset(java.util.BitSet subset, Instance holdOut, boolean retrain)`             Evaluates a subset of attributes with respect to a single instance. |
| `abstract  double` | `evaluateSubset(java.util.BitSet subset, Instances holdOut)`             Evaluates a subset of attributes with respect to a set of instances. |

**Methods inherited from class weka.attributeSelection.ASEvaluation**

`buildEvaluator, forName, getCapabilities, getRevision, makeCopies, postProcess`

**Methods inherited from class java.lang.Object**

`equals, getClass, hashCode, notify, notifyAll, toString, wait, wait, wait`

**Methods inherited from interface weka.attributeSelection.SubsetEvaluator**

`evaluateSubset`

**Constructor Detail**

### HoldOutSubsetEvaluator

```
public HoldOutSubsetEvaluator()
```


**Method Detail**

### evaluateSubset

```
public abstract double evaluateSubset(java.util.BitSet subset,
                                      Instances holdOut)
                               throws java.lang.Exception
```

:   Evaluates a subset of attributes with respect to a set of instances.

    :   **Parameters:**: `subset` - a bitset representing the attribute subset to be evaluated: `holdOut` - a set of instances (possibly seperate and distinct from those use to build/train the evaluator) with which to evaluate the merit of the subset **Returns:**: the "merit" of the subset on the holdOut data **Throws:**: `java.lang.Exception` - if the subset cannot be evaluated

---


### evaluateSubset

```
public abstract double evaluateSubset(java.util.BitSet subset,
                                      Instance holdOut,
                                      boolean retrain)
                               throws java.lang.Exception
```

:   Evaluates a subset of attributes with respect to a single instance.

    :   **Parameters:**: `subset` - a bitset representing the attribute subset to be evaluated: `holdOut` - a single instance (possibly not one of those used to build/train the evaluator) with which to evaluate the merit of the subset: `retrain` - true if the classifier should be retrained with respect to the new subset before testing on the holdOut instance. **Returns:**: the "merit" of the subset on the holdOut instance **Throws:**: `java.lang.Exception` - if the subset cannot be evaluated


---


|  |  |  |
| --- | --- | --- |
| **Overview** \| **Package** \| **Class** \| **Tree** \| **Deprecated** \| **Index** \| **Help** \| **Weka's home** | |  |
| **PREV CLASS**   **NEXT CLASS** | **FRAMES**    **NO FRAMES**     **All Classes** |  |
| SUMMARY: NESTED \| FIELD \| CONSTR \| METHOD | DETAIL: FIELD \| CONSTR \| METHOD |  |


---
